# Academy of Medicine and Surgery in the City of New-York

**Published:** 1847-02

**Authors:** 


*Academy of Medicine and Surgery in the city of New-York.*—A movement is now being made in the city of New-York to organize an association under the above title, for the promotion of professional and social intercourse, the elevation of the character of the profession, and the enforcement of Medical Ethics, The enterprise is sustained by the leading members of the profession, and appears to have enlisted general interest. It is contemplated to erect a suitable building for the meetings of the Academy. We rejoice in this striking evidence of the harmony and spirit which prevail among our brethren of the city of New-York. The resources and the ability are there to make the Emporium of the country what, in a medical point of view, it may be — the Paris of America.

We contemplate great results from an association which will unite all in the interests of a common science and a true brotherhood. May no untoward circumstances prevent the consummation of all that the academy promises to effect!

Since the above was written we find in the N. Y. Med. and Surg. Reporter the *constitution* adopted by the Academy, which is as follows:

ARTICLE 1. This Association shall be called the "NEW-YORK ACADEMY OF MEDICINE," and be composed of Resident and Corresponding Fellows.

ART, II. The object of the Academy shall be:—

1st. The separation of Regular and Irregular Practitioners.

2d. The association of the Profession Proper for purposes of mutual recognition and fellowship.

3d. The promotion of the character, interests, and honor of the fraternity, by maintaining the union and harmony of the regular profession of the City and its vicinity, and aiming to elevate the standard of Medical Education.

4th, The cultivation and advancement of the Science, by onr united exertions for mutual improvement, and our contributions to Medical Literature.

ART. III. The Resident Fellows shall be Regular Prectitioners of Medicine or Surgery in the city of New-York or its vicinity; shall be proposed by a Fellow of the Academy to the Committee on Admissions, which shall satisfy itself of the regular standing of the candidate, by credentials or otherwise, and upon its recommendation he may be admitted by vote of the Academy at a regular meeting. A residence of three years in this city or vicinity shall be necessary to eligibility in the Fellowship of the Academy.

ART. IV. No Proprietor or Vender of any patent or secret remedy or medicine, or any Empirical or Irregular Practitioner, shall either be admitted to, or retained in, the Fellowship of this Academy.

ART. V. Corresponding Fellows may be elected on the nomination of the Committee on Admissions, which shall vouch for their being duly qualified practitioners; but the votes of three-fourths of the Fellows present, at a regular meeting shall be necessary for such election. The number of Corresponding fellows shall be limited to one hundred.

ART. VI. The Officers of this Academy shall be, a President, four Vice-Presidents, a Recording Secretary, two Corresponding Secretaries, designated for Domestic and Foreign Correspondence; a Treasurer, and a Librarian; who shall be elected annually by ballot at the regular meeting in January. They shall severally perform the duties indicated by the title of their respective offices.

ART. VIII. The President shall appoint, immediately after his election, the following Standing Committees, each of which shall consist of five Resident Fellows:—

1st. A Committee on Admissions.
2d. A Committee on Finance.
3d. A Committee on Medical Ethics.
4th. A Committee on Publication.
5th, A Council of Appeal.

ART. VIII. Alterations of this Constitution shall not be made except at a meeting subsequent to that at which such alteration shall have been proposed, in writing.

